# Left gastric artery embolization for recurrent massive intraluminal postoperative bleeding after revisional laparoscopic one anastomosis gastric bypass surgery

**DOI:** 10.1093/jscr/rjae070

**Published:** 2024-05-03

**Authors:** Ivan Kukeev, Elchanan Quint, Gilbert Sebbag, Oleg Dukhno

**Affiliations:** Department of General Surgery B, Soroka University Medical Center, Ben-Gurion University of the Negev, Yitzhack I. Rager Blvd 151, PO Box 151, Beer Sheva 84101, Israel; Department of General Surgery B, Soroka University Medical Center, Ben-Gurion University of the Negev, Yitzhack I. Rager Blvd 151, PO Box 151, Beer Sheva 84101, Israel; Department of General Surgery B, Soroka University Medical Center, Ben-Gurion University of the Negev, Yitzhack I. Rager Blvd 151, PO Box 151, Beer Sheva 84101, Israel; Bariatric Surgery Unit, Department of General Surgery B, Soroka University Medical Center, Ben-Gurion University of the Negev, Yitzhack I. Rager Blvd 151, PO Box 151, Beer Sheva 84101, Israel

**Keywords:** bariatric surgery, postoperative bleeding, embolization, gastric bypass

## Abstract

Laparoscopic one-anastomosis gastric bypass (LOAGB) has gained popularity as safe weight-reduction procedure. Bleeding is the common postoperative complication. We present a successful treatment of recurrent bleeding after LOAGB by embolization of the left gastric artery (LGA) and later development of necrotizing pancreatitis. A 41-year-old patient with previous bariatric surgeries undergone LOAGB surgery with development of massive intraluminal bleeding in the postoperative period. Attempts of unsuccessful endoscopic treatment were done and the bleeding was stopped by LGA embolization. In the post-embolization period, the patients developed necrotizing pancreatitis. Postoperative bleeding is the serious complications of the bariatric LOAGB procedure. Transcatheter Arterial Embolization (TAE) is the possible treatments after unsuccessful endoscopic attempts to stop the bleeding. The technical and clinical success rates of TAE in post-gastrectomy bleeding are 100 and 79%, respectively. TAE can be successfully used to stop obstinate recurrent postoperative bleeding after a LOAGB procedure.

## Introduction

Laparoscopic one-anastomosis gastric bypass (LOAGB) is a relatively new bariatric procedure for morbid obesity that has increased in use internationally as a low-risk, rapid, and effective procedure. The most common reported postoperative complications are bleeding, leakage, and ulcers. Major bleeding is the most common complication requiring endoscopic intervention or revision surgery, whereas intraluminal acute bleeding is a rare complication [1].

We present a successful left gastric artery (LGA) embolization for recurrent massive intraluminal bleeding after a LOAGB procedure with the development of post-embolization necrotizing pancreatitis. Informed consent was obtained from the patient for publication.

## Presentation

A 41-year-old patient with very severe obesity (BMI 51.2) was admitted for an elective LOAGB procedure. The patient’s medical history was hypertension, obstructive sleep apnoea, dyslipidaemia, and persistent back pain. His surgical history was three laparoscopic adjustment gastric band procedures, with the last operation 10 years previously. Due to weight regain, the patient underwent laparoscopic removal of the gastric band and one-anastomotic gastric bypass in one step. After removal of the gastric band and surrounding fibrotic tissue, a new gastric pouch was created using an endo-stapler (Signia stapler, Medtronic, USA). A stapled gastroenterotomy was done and the enterotomy was closed using two layers of V-lock suture (Medtronic, USA). Fibrin sealant (EVICEL, Ethicon, USA) was added at the staple line for additional haemostasis. An abdominal drain was left in the anastomosis area. No intraoperative complication was noted.

**Figure 1:**
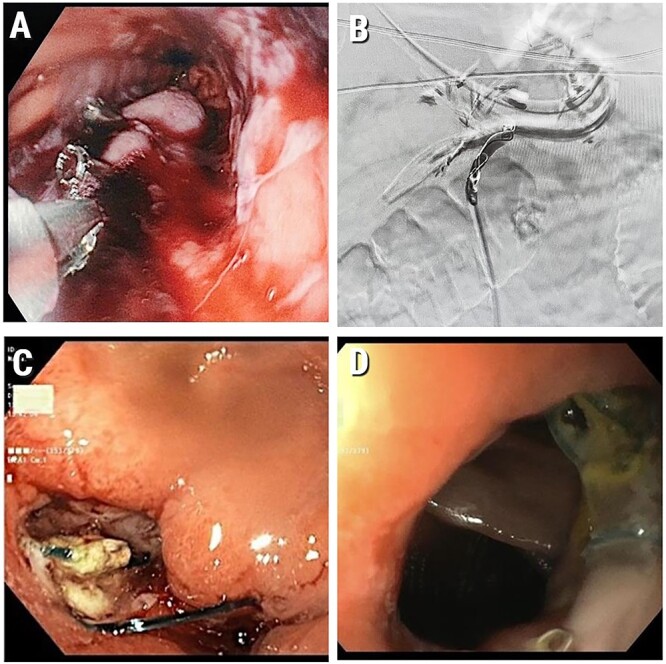
(A) Intraluminal gastric bleeding stopped with clipping at the stapler line; (B) Embolized LGA; (C) Gastroscopy at POD 25. A marginal ulcer with a clean base; (D) Healed marginal anastomotic ulcer (at gastroscopy 1.5 months post operation). We have obtained informed consent from patient to publish this manuscript.

A few hours post surgery, the patient presented with severe haematemesis and haemorrhagic shock (consistent tachycardia of 120 beats per minute). The patient’s haemoglobin level decreased from 12 to 7 g/dL. The patient was immediately taken to the operating room where no blood was found during an examination of the abdominal cavity, but there was a haematoma in the area of the gastrojejunostomy. The staple lines were sutured with the addition of haemostatic gel (SURGIFLOW, Ethicon, USA). After this second operation, he was admitted to the intensive care unit (ICU) and received two doses of packed cells. In the ICU, the patient’s condition did not improve as expected. At this point, based on a working hypothesis of intra-lumen bleeding, an urgent gastroscopy was done and revealed blood clots at the stapler line with bleeding from the lower oesophagus, which was stopped by clipping (Fig. 1A).

Due to later rebleeding, the patient underwent a repeated gastroscopy, where numerous areas of bleeding were found along the entire gastric stump and additional haemostasis was performed by cauterization and clipping. Despite this, the intra lumen bleeding continued. Because of the failed surgical and gastroscopic attempts to stop the bleeding, an angio-embolization of the LGA with embozene microspheres (USA) and coils (Boston Scientific Corporation, USA) was performed (Fig. 1B).

After receiving the packed cells as mentioned above, the patient’s haemoglobin level stabilized at 8.7 g/dL. The patient started total parenteral nutrition (TPN) and a high dose of proton pump inhibitors (PPI). At postoperative day (POD) 5, the patient had a fever (up to 38°C) with elevated inflammatory markers (WBC 30.8 × 10^9^/L, CRP – 40.17 mg/dL) as well as a slight elevation of pancreatic enzymes (amylase – 235 U/L, lipase – 176 U/L). The patient started with empiric IV antibiotic therapy Piperacillin/Tazobactam (Tazocin) and Fluconazole. The patient underwent an abdominal CT scan at POD 6, which revealed oedematous pancreatitis. After another 5 days of the above treatment the patient’s parameters stabilized, blood markers returned to normal values, the patient began to eat and was later discharged from our department.

Ten days later (POD 21), the patient returned to the ED with signs of sepsis and acute kidney injury. Haemodynamic parameters were as follows: blood pressure 143/75, pulse 132 (blood tests – Hb 9.3 g/dL, WBC 33.15 × 10^9^/L, neutrophils, abs. – 29.35 cells/mm^3^, creatinine – 2.58 mg/dL, amylase – 229 U/L, lipase – 281 U/L, CRP – 16.83 mg/dL). A few days later, he underwent another CT that revealed changes in the pancreatic tissue and fluid collections. Necrotizing pancreatitis was diagnosed.

The patient resumed conservative treatment with IV antibiotic cover with Piperacillin/Tazobactam (Tazocin) and Fluconazole beside nutritional treatment with TPN. Despite these measures, the patient had a fever, with a temperature up to 39.5°C. Based on the assumption of a possible leakage of the anastomosis, the patient underwent gastroscopy at POD 25. Repeated gastroscopy revealed ulcers in the anastomotic area (marginal ulcer) without bleeding or leakage (Fig. 1C). Gastric mucosa had no signs of ischaemia.

Over the next 2 weeks, the patient’s condition gradually improved and he was finally discharged with PPI PO and parenteral support. On CT a month and a half after the index operation partial absorption of the pancreas collections was seen. On gastroscopy, a marginal ulcer at the healing stage with no evidence of ischaemic lesions was observed (Fig. 1 D).

In the 2 months after the index surgery the patient was independent, able to socialize, and decreased in weight by 27 kg (EWL– 28%).

## Discussion

One-anastomosis gastric bypass (OAGB) was devised in 1997 by Robert Rutledge as a restrictive and malabsorptive bariatric procedure and is the fourth most performed bariatric operation in Europe and in the Asia/Pacific area. Several authors have demonstrated the effectiveness of this surgical technique in terms of both weight loss and the resolution of comorbidities. Early complications include bleeding, infection, leak, abscess, respiratory complications (atelectasis, pneumonia, aspiration, and pulmonary embolism), urinary infection, and urine retention. In his series of 6400 OAGB patients, Rutledge found that the early complication rate was ~5.0%. Major bleeding occurs in 0.2–28.6% of cases and requires endoscopic intervention or revision surgery [1].

Susmallian and colleagues determined the factors that influence bleeding during the course of bariatric surgery. Their retrospective study included 8544 cases that underwent bariatric surgery from 2013 to 2016. Bleeding was the most frequent complication that occurred in 122 (1.3%) patients. The factors that influence the occurrence of perioperative bleeding in bariatric surgery are hypertension, chronic lung disease, age > 45 years, arrhythmia, and the surgeon’s skills [2]. Revision procedures are increasing in frequency, but as a type of surgery they are known to be less effective and have a higher complication rate [12]. The 2016 IFSO stated that revision surgery accounted for 7.4% of all bariatric procedures globally. Over 50% of patients that underwent a laparoscopic adjustable gastric banding procedure required revision surgery.

Bleeding after an LOAGB procedure can originate at one of three potential staple lines: the long gastric pouch, the excluded stomach, and the gastrojejunal anastomosis. Non-alcoholic fatty liver, coagulopathy, hypertension, and super obesity increase the risk of bleeding and should be carefully evaluated preoperatively. The stomach has a rich vascular supply provided by a collateral arterial plexus. A study using an animal model reported that ligation of up to 95% of the arterial supply to the stomach did not cause perfusion defects in the gastric mucosa. Blood supply to the gastric pouch after the LOAGB originates from the LGA, and in some cases from the left subphrenic artery and undivided branches of short gastric vessels. Because of the abundant blood supply in the stomach, cessation of bleeding by acting on the LGA can lead to ischaemic damage of the stomach wall and anastomotic area. Therefore, this ‘last resort’ decision should be used with care. In our case we performed LGA embolization due to recurrent intraluminal bleeding, which did not stop after two interventional gastroscopies and surgical staple line over-suturing. We also suggest that the development of necrotizing pancreatitis in our patient was a complication of the embolization procedure and may be related to temporary gastroduodenal artery blockade during intravascular access.

Based on evidence in the literature, adequate prevention of bleeding involves reinforcing the staple line (buttress and Lembert’s suture, glue), choosing an adequate cartridge height, and controlling haemostasis with low intra-abdominal pressure (≤10 mm/Hg). The American Gastroenterological Association guidelines for endoscopic therapies for non-variceal upper gastrointestinal bleeding recommend a second attempt at endoscopic haemostasis (additional thermal therapy and/or clipping) in patients with rebleeding following endoscopic therapy [3]. The main options in cases of endoscopic failure with rebleeding/refractory bleeding are transcatheter arterial embolization (TAE) and surgery. TAE has been shown to be equally effective with lower 30-day mortality rates compared with surgical intervention. The advantage of TAE is that it may be used in coagulopathy settings and patients who are not eligible for surgery [4]. TAE demonstrated fewer complications in comparison with surgery, but higher rebleeding rates [4]. Han and colleagues published a study about the feasibility and safety of TAE in the management of post-gastrectomy arterial bleeding. Between 2004 and 2015, 24 patients underwent TAE. The technical and clinical success rates of TAE in post-gastrectomy bleeding are 100 and 79%, respectively [5]. The clinical success rate was lower due to procedure related complication resulting in the death of three patients (1-bowel perforation; 2-infarctions of spleen and remnant stomach). Embolization of multiple/large duodenal ulcers, coagulopathy, or multi-organ failure contribute to clinical failures. The most effective embolization can be achieved with bleeding site pre-localization (anatomical location/haemoclip). The most common complications of TAE are groyne haematomas (3–17%) and contrast-related renal failure (0.04–12.7%). For optimal clinical outcomes in refractory bleeding, a multidisciplinary approach is required, involving weighing the risk and benefits of each intervention on a case-by-case basis.

## Conclusion

Early postoperative massive bleeding is a challenge for the surgeon. Using the correct tactics and sequence of actions helps to avoid unnecessary complications. Embolization of the main feeding artery is a possible option in the management of acute bleeding and should be considered in cases where it is impossible to stop the bleeding by other less ‘aggressive’ methods. The possibility of developing ischaemic disorders and repeated resectional interventions must be carefully assessed in each case.
